# Blood autoantibody and cytokine profiles predict response to anti-tumor necrosis factor therapy in rheumatoid arthritis

**DOI:** 10.1186/ar2706

**Published:** 2009-05-21

**Authors:** Wolfgang Hueber, Beren H Tomooka, Franak Batliwalla, Wentian Li, Paul A Monach, Robert J Tibshirani, Ronald F Van Vollenhoven, Jon Lampa, Kazuyoshi Saito, Yoshiya Tanaka, Mark C Genovese, Lars Klareskog, Peter K Gregersen, William H Robinson

**Affiliations:** 1 Department of Medicine, Division of Immunology & Rheumatology, Stanford University, 269 Campus Drive, mail code 5166, Stanford, CA 94305, USA; 2 GRECC, VA Palo Alto Health Care Systems, 3801 Miranda Ave, mailstop 154R, Palo Alto, CA 94304, USA; 3 Feinstein Institute of Medical Research, North Shore LIJ Health System, 350 Community Drive, Manhasset, NY 11030, USA; 4 Joslin Diabetes Center, One Joslin Place, Boston, MA 02215, USA; 5 Brigham and Women's Hospital, 75 Francis Street, Boston, MA 02115 USA; 6 Department of Statistics, 390 Serra Mall, Stanford University, Stanford, CA 94305, USA; 7 Karolinska Institutet, Building D2:02, SE-171 76 Stockholm, Sweden; 8 First Department of Internal Medicine, University of Occupational & Environmental Health, 1-1 Iseigaoka, Yahata-nishi, Kitakyushu 807-8555, Japan

## Abstract

**Introduction:**

Anti-TNF therapies have revolutionized the treatment of rheumatoid arthritis (RA), a common systemic autoimmune disease involving destruction of the synovial joints. However, in the practice of rheumatology approximately one-third of patients demonstrate no clinical improvement in response to treatment with anti-TNF therapies, while another third demonstrate a partial response, and one-third an excellent and sustained response. Since no clinical or laboratory tests are available to predict response to anti-TNF therapies, great need exists for predictive biomarkers.

**Methods:**

Here we present a multi-step proteomics approach using arthritis antigen arrays, a multiplex cytokine assay, and conventional ELISA, with the objective to identify a biomarker signature in three ethnically diverse cohorts of RA patients treated with the anti-TNF therapy etanercept.

**Results:**

We identified a 24-biomarker signature that enabled prediction of a positive clinical response to etanercept in all three cohorts (positive predictive values 58 to 72%; negative predictive values 63 to 78%).

**Conclusions:**

We identified a multi-parameter protein biomarker that enables pretreatment classification and prediction of etanercept responders, and tested this biomarker using three independent cohorts of RA patients. Although further validation in prospective and larger cohorts is needed, our observations demonstrate that multiplex characterization of autoantibodies and cytokines provides clinical utility for predicting response to the anti-TNF therapy etanercept in RA patients.

## Introduction

Rheumatoid arthritis (RA) is a prototypical systemic autoimmune disease that affects 1% of the world population. TNF antagonists have become the most widely used biological therapies for patients with RA [1]. Based on criteria to quantify response to therapy with disease-modifying anti-rheumatic drugs [2], 30 to 50% of patients achieved an ACR50 or greater response to anti-TNF therapies in sentinel clinical trials [3-5]. American College of Rheumatology (ACR) response criteria are a composite index of measures indicative of the percentage improvement over baseline that was achieved by an individual patient while on treatment for at least 12 weeks, with ACR20 the primary measure of efficacy [6]. Clinical trials, however, generally focus on homogeneous populations that frequently include more severely ill patients who are more likely to show statistically significant improvement over placebo [7,8]. In contrast, large observational studies of the mixed populations of RA patients typical of clinical practice indicate that longer term response rates to anti-TNF therapies may be considerably lower than those reported in these landmark clinical trials [7-10].

Great need exists for molecular biomarkers for the prediction of response to anti-TNF therapies, and a number of candidate markers are currently under investigation, including genetic and protein markers [11]. RA is associated with the production of multiple autoantibody specificities and the dysregulation of multiple cytokines, which are both present in the serum proteome in RA patients [12]. Since cytokines and potentially autoantibodies contribute to the pathogenesis of RA, we reasoned that characterization of spectra of serum autoantibodies and cytokines, rather than characterizing the entire serum proteome, might yield tractable biomarkers for guiding anti-TNF therapy in RA.

We previously reported the development of antigen microarrays and application of these arrays to characterize autoantibody phenotypes associated with a variety of autoimmune diseases [13]. We further developed RA antigen microarrays, and applied these arrays to identify autoantibody profiles that molecularly stratify RA patients into clinical subgroups [14]. We have also demonstrated the utility of blood cytokine profiling to subclassify patients with early RA, and demonstrated an association of elevated blood levels of the proinflammatory cytokines TNF, IL-1β, IL-6, IL-13, IL-15 and granulocyte- macrophage colony-stimulating factor with autoantibody targeting of citrullulinated antigens [12].

**Figure 1:**
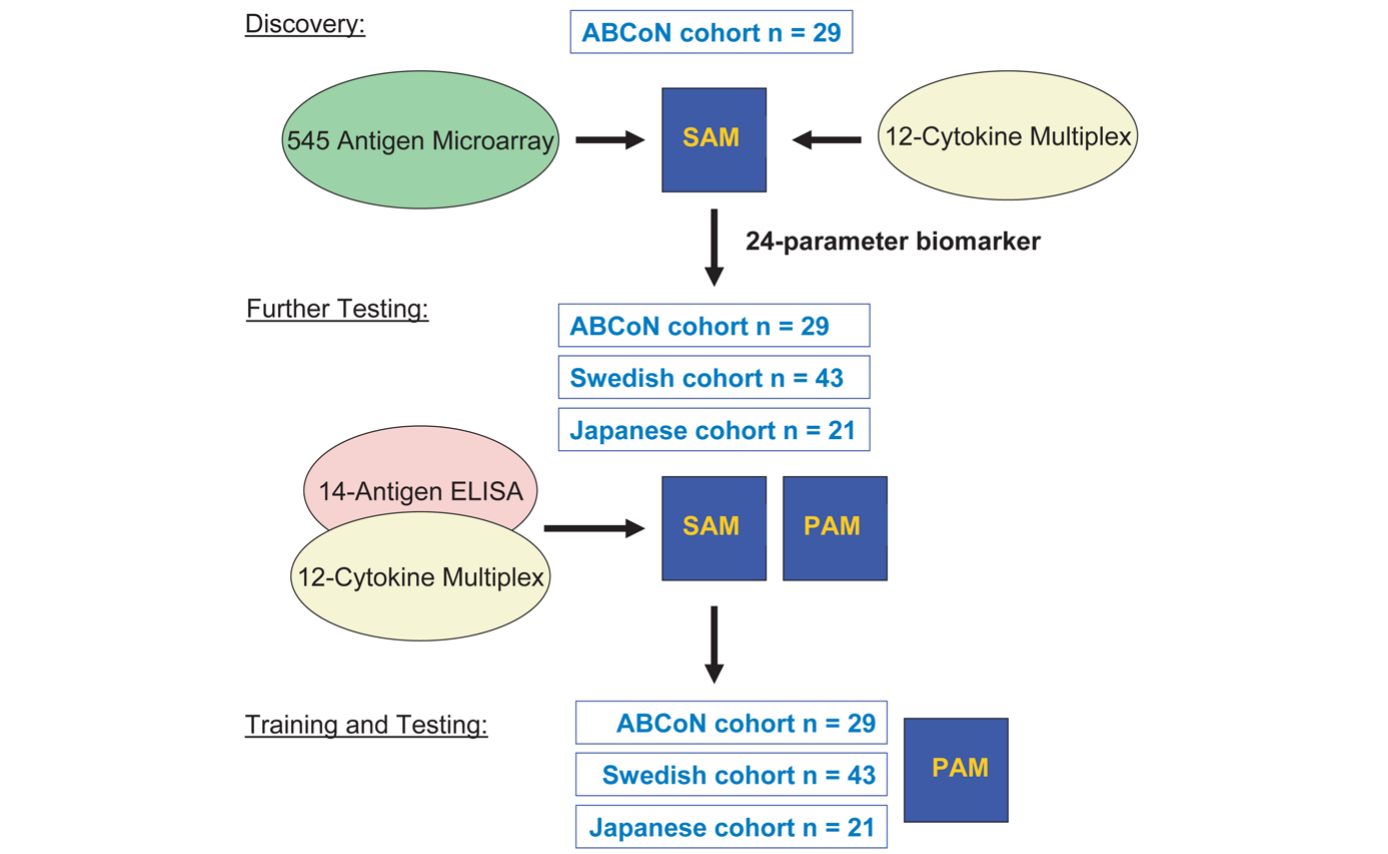
Workflow of experiments and types of analysis. Upper panel: in the discovery steps, synovial antigen microarrays and multiplex cytokine assays were employed to determine candidate molecules that are differentially expressed in pretreatment sera of etanercept responders (≥ ACR50) and nonresponders (< ACR20). Multiple array experiments were performed, each followed by significance analysis of microarrays (SAM) to identify the highest-scoring discriminators. Middle panel: further testing was performed with three independent cohorts using standard ELISAs, followed by prediction of response in three cohorts of etanercept-treated patients using prediction analysis of microarrays (PAM). Bottom panel: for training and testing, PAM was used to identify the best discriminators (training step; which identified a 24-biomarker panel) and then the utility of these discriminators for predicting response to etanercept was determined (testing). ACR, American College of Rheumatology response.

In the present report, we describe application of a multi-step proteomics approach using RA antigen arrays and cytokine arrays to discover and validate a multivariable biomarker for prediction of response to the anti-TNF therapy etanercept, using sera derived from three independent cohorts of patients with RA. The workflow of the studies is outlined in Figure 1.

## Materials and methods

### Patient sera

Pretreatment sera from three cohorts of patients with the diagnosis of RA based on the ACR classification criteria [15], who were initiated on therapy with the anti-TNF therapy etanercept (Enbrel^®^, Amgen, Thousand Oaks, CA, USA), were analyzed using synovial antigen microarrays (except for the third cohort), ELISAs, and a multiplex 12-cytokine bead assay. The cytokines assayed were selected based on previous screening studies using a 22-cytokine assay [12].

The three cohorts included 29 Caucasian patients from the ABCoN cohort of the North American Rheumatoid Arthritis Consortium (US cohort), 43 Caucasian patients seen at Swedish tertiary care centers and collected through the Karolinska-lead EiRA initiative (Swedish cohort), and 21 Japanese patients (Japanese cohort). The patients' demographic, clinical and serologic characteristics are summarized in Table 1 and Additional data file 1. All patients signed informed consent and all sera were collected under and in accordance with Institutional Review Board-approved protocols at each institution.

**Table 1:** Demographic, clinical and serologic characteristics of the three cohorts

Cohort	Parameter
US-based (ABCoN)	
Age (years)	50 (35 to 78)
Female sex	23 (79)
CCP2	56.6 (1.2 to 500)
CRP (mg/l)	60 (2 to 79)
ACR response < 20	15 (51.7)
ACR response > 50	14 (48.3)
Shared epitope present	16/24 (66)
Disease duration (months)	96 (12 to 396)
Swedish	
Age (years)	54 (32 to 77)
Female sex	34 (79)
CCP2	n.d.
CRP (mg/l)	37.5 (10 to 166)
ACR response < 20	19 (44.2)
ACR response > 50	24 (55.8)
Shared epitope present	n.d.
Disease duration (months)	108 (1 to 464)
Japanese	
Age (years)	56 (24 to 76)
Female sex	20 (95%)
CCP2	n.d.
Rheumatoid factor (units)	66.9 (14.9 to 1,675)
CRP (mg/l)	55 (33 to 75)
ACR response < 20	9/21 (29.0)
ACR response > 70	12/21 (38.7)
Shared epitope present	n.d.
Disease duration (months)	151 (11 to 444)

Data presented as median (range) or *n *(%). ACR, American College of Rheumatology; CCP, cyclic citrullinated peptide; n.d., not determined.

Blood samples were obtained at baseline (pretreatment sample) and at least 3 months after initiation of therapy with etanercept. Analysis of the pretreatment samples was performed for the present study. Response to therapy with etanercept was assessed at least 3 months after etanercept was started, based on the ACR criteria for improvement [2]. All samples were immediately aliquoted upon receipt at the Stanford research laboratory, and separate aliquots were used for each assay to minimize the effects of additional freeze- thaw cycles.

### Cytokine assay

All cytokine measurements were performed using the Luminex ×200 platform, following a previously described optimized assay protocol [12]. Briefly, to minimize potential false-positive elevations of cytokine measurements due to rheumatoid factor and other heterophilic antibodies that can cross-link the capture and detection antibodies, HeteroBlock^® ^was added to achieve a final concentration of 3 μg/ml, as previously described in detail [12]. For the studies performed herein, we utilized a custom 12-plex human cytokine FLEX^® ^kit (Upstate, Millipore, Billerica, MA, USA) that included beads specific for TNFα, IL-1α, IL-1β, IL-6, IL12p40, IL-12p70, IL-15, granulocyte- macrophage colony-stimulating factor, fibroblast growth factor-2 (FGF-2), monocyte chemoattractant protein-1 (MCP-1), eotaxin, and IFNγ-inducible protein 10.

### RA antigen microarrays

The production of RA antigen microarrays was previously described in detail [13,14]. Briefly, more than 500 peptides and proteins representing candidate autoantigens in RA were printed at 0.2 μg/μl onto derivatized Epoxy ArrayIt^® ^microscope slides (ArrayIt^®^; TeleChem International Inc., Sunnyvale, CA, USA) using a robotic arrayer. The arrays were blocked and probed with sera at 1:200 dilutions, and bound serum autoantibodies detected using Cy3-conjugated goat anti-human IgG secondary antibodies (Jackson ImmunoResearch Laboratories, Inc. West Grove, PA, USA). Probed arrays were scanned with a GenePix 4000B scanner (MDS Analytical Technologies, Sunnyvale, CA, USA), and antibody reactivities quantified using GenePix Pro 5.0 software.

Peptides cfc(48–65)cit1 (shown in Figure 1) and peptides cfc(48–65)cit2 (listed in Table 2) are identical except for a difference in the degree of citrullination: cfc(48–65)cit1 has one arginine residue citrullinated, and cfc(48–65)cit2 has two arginine residues citrullinated.

**Table 2:** Candidate biomarkers identified in array screening experiments, subsequently used for training and cross-validation in PAM

Variable	Peptide sequence	SwissProt ID
Autoantigens		
Acetyl-calpastatin(184–203)	Ace-DPMSSTYIEELGKREVTIPP- [NH]_2_	[Swiss-Prot:P20810]
Apolipoprotein E(277–296)cit	A [cit]LKSWFEPLVEDMQ [cit]QWAG	[Swiss-Prot:P02649]
Cfc(48–65)cit2	TIHAHPGS [cit]RGG [cit]HGYHH	[Swiss-Prot:P20930]
Clusterin(170–188)	QTHMLDVMQDHFSRASSID	[Swiss-Prot:P10909]
Clusterin(334–353)cit2	AE [cit]LT [cit]KYNELLKSYQWKML	[Swiss-Prot:P10909]
Fibrinogen A(616–635)cit3	THSTK [cit]GHAKS [cit]PV [cit]GIHTS	[Swiss-Prot:P02671]
Fibromodulin(246–265)	LEQLYMEHNNVYTVPDSYFR	[Swiss-Prot:Q06828]
H2B/e(1–20)	MPEPVKSAPVPKKGSKKAIN	[Swiss-Prot:Q16778]
HSP60(287–297)	VLNRLKVGLQV	[Swiss-Prot:P10809]
Osteoglycin(176–195)	NQLLKLPVLPPKLTLFNAKY	[Swiss-Prot:P20774]
Serine protease 11(433–452)	VIISINGQSVVSANDVSDVI	[Swiss-Prot:Q92743]
Biglycan(247–266)	EDLLRYSKLYRLGLGHNQIR	[Swiss-Prot:P21810]
Cartilage oligomeric matrix protein(453–472)	NSAQEDSDHDGQGDACDDDD	[Swiss-Prot:P49747]
Fibrinogen cit	n.a.	[Swiss-Prot:P02671]
Cytokines and chemokines		
Eotaxin	n.a.	[Swiss-Prot:P51671]
Fibroblast growth factor-2	n.a.	[Swiss-Prot:P09038]
Granulocyte- macrophage colony-stimulating factor	n.a.	[Swiss-Prot:P04141]
IL-12p40	n.a.	[Swiss-Prot:P29460]
IL-12p70	n.a.	[Swiss-Prot:P29459]
IL-15	n.a.	[Swiss-Prot:P40933]
IL-1α	n.a.	[Swiss-Prot:P01583]
IL-1β	n.a.	[Swiss-Prot:P01584]
IL-6	n.a.	[Swiss-Prot:P05231]
IFNγ-inducible protein 10	n.a.	[Swiss-Prot:P02778]
TNFα	n.a.	[Swiss-Prot:P01375]
Monocyte chemoattractant protein-1	n.a.	[Swiss-Prot:P13500]

n.a., not applicable; PAM, prediction analysis of microarrays.

### Enzyme-linked immunsorbent assay

Peptides and fibrinogen protein from human plasma (Calbiochem, Gibbstown, NJ, USA) were coated onto medium-binding 96-well flat-bottom polystyrene plates (Costar, Corning Inc, Corning, NY, USA) at 1 μg peptide/ml at 4°C overnight. Plates were then washed and blocked for 1 hour at room temperature using 5% dry milk in PBS, and incubated for 90 minutes using serum at 1:200 dilution in PBS. Horseradish peroxidase-conjugated anti-human IgG secondary antibodies (horseradish peroxidase-conjugated goat anti-human IgG, Fcγ-fragment specific; Jackson Immuno Research Laboratories Inc., West Grove, PA, USA) were used at 1:20,000 dilutions, and bound autoantibodies were detected by chemoluminescence (Onestep^® ^TMB ELISA; Pierce, Rockford, IL, USA).

### Data analysis

The data analysis was performed using significance analysis of microarrays (SAM) (version 1.21) and prediction analysis of microarrays (PAM) (version 1.23), and the hierarchical clustering software Cluster^® ^and TreeView^® ^[16], as described previously [14,17]. PAM uses internal cross-validation by which 90% of the training samples are randomly selected 10 times, followed by one-by-one class prediction of the remaining 10% of samples, thus identifying classification errors and overfitting [18]. PAM was used in the training, cross-validation and prediction analyses described. The general-purpose statistical package R has also been used for the analysis [19].

Non-normalized datasets were used for all analyses in the article. Although this approach limits the ability to detect differences in the low signal intensity range, the rationale for this approach is based on the observation that high-level reactivities became significantly distorted when *z-*normalization procedures were applied.

## Results

### Expansion of RA antigen microarrays for profiling autoantibodies in RA sera

To further develop previously-described RA autoantigen microarrays [14], we expanded the number of peptide and protein autoantigens on the arrays. The arrays used in the experiments described herein were 2,180-feature arrays that include > 540 proteins and peptides, representing the following candidate RA antigens: biglycan; decorin; fibromodulin; clusterin; osteoglycin; fibrinogen; type I, type II, and type V collagens; vimentin; filaggrin; serine protease 11; apolipoprotein E; calpastatin; glucose-6-phosphate isomerase; heat shock proteins HSP60, HSP70, HSP90, and BiP; hnRNP-A2/B1; histones H2A and H2B; and cartilage oligomeric matrix protein (COMP), vitronectin and fibronectin. Candidate antigens were selected based on literature searches and screening experiments. Briefly, for the antigen screening experiments, immune complexes were isolated from RA cartilage and synovial tissues, and the protein antigens contained in the synovial immune complexes were identified by mass spectrometry (PM, manuscript in preparation). Identified proteins were, when available, purchased from commercial sources and peptides representing the candidate antigens synthesized for printing on arrays. Overlapping 20-amino acid peptides, both native and containing citrulline substitutions, were synthesized on a commercial custom peptide synthesis platform using Fmoc chemistry (PepScreen^®^; Sigma Genosys, St Louis, MO, USA).

**Figure 2:**
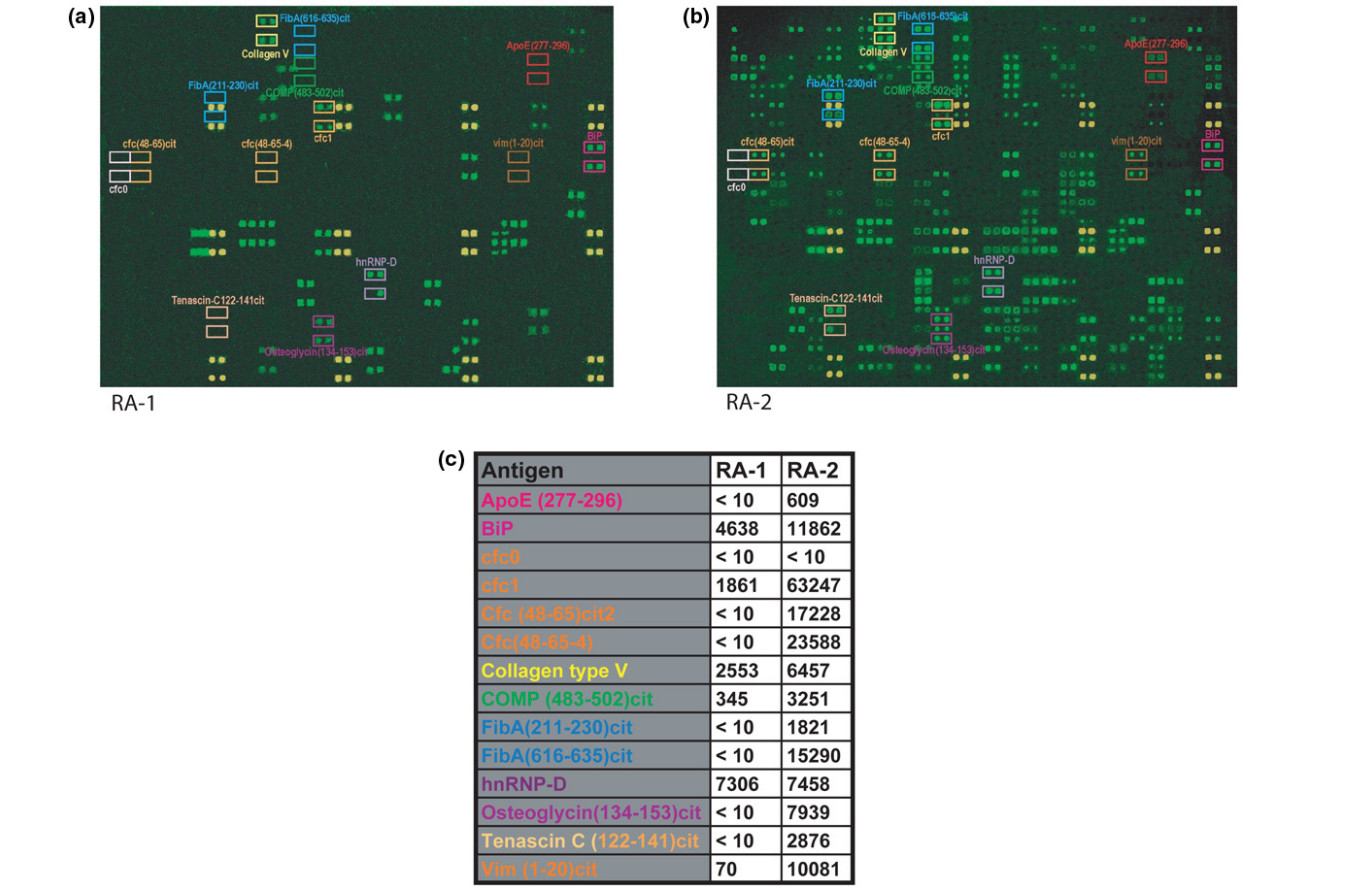
Rheumatoid arthritis antigen microarrays. Rheumatoid arthritis (RA) antigen microarrays were used for autoantibody profiling of sera derived from patients with RA prior to initiation of etanercept therapy. **(a), (b) **Array results from two representative RA patients. Yellow features are false-colored features utilized for array orientation, while green features represent autoantibody reactivities. Selected autoantibody reactivities are highlighted in colored boxes. **(c) **Quantification of the highlighted features. ApoE, apolipoprotein E; COMP, cartilage oligomeric matrix protein.

In line with and expanding earlier results using smaller 225-antigen arrays, we observed on the > 540-antigen arrays specific and differential serum autoantibody reactivities to COMP, clusterin, osteoglycin, apolipoprotein E, histones H2A and H2B, serine protease 11, and other candidate antigens. Representative array images of antibody reactivities are shown for sera from two different patients with RA; one patient that exhibited low serum autoantibody reactivity (Figure 2a) and another that exhibited high serum autoantibody reactivity (Figure 2b). A selection of antigen targets that were differentially recognized by serum antibodies in patients RA1 and RA2 are highlighted in the figures in colored boxes (Figure 2a, b). Selected array reactivities include citrullinated peptides (cfc1, reactive with anti-cyclic citrullinated peptide antibody-positive RA serum) and native control peptides (cfc0, no reactivity with RA sera). Features were normalized to IgG/M, and quantification of the highlighted features is summarized in Figure 2c.

To determine the correlation of antigen array and ELISA results from a subset of peptide antigens, several native and citrulline-substituted peptides were tested in ELISA experiments. We observed moderate to strong correlations for these peptide antigens, with correlation coefficients *R*^2 ^ranging from 0.49 for clusterin(386–405)cit to > 0.92 for hFibA(211–230)cit (see Additional data file 2).

### Exploratory profiling using samples from the ABCoN cohort

#### Pretreatment autoantibody profiles differentiate anti-TNF therapy responders from nonresponders

**Figure 3:**
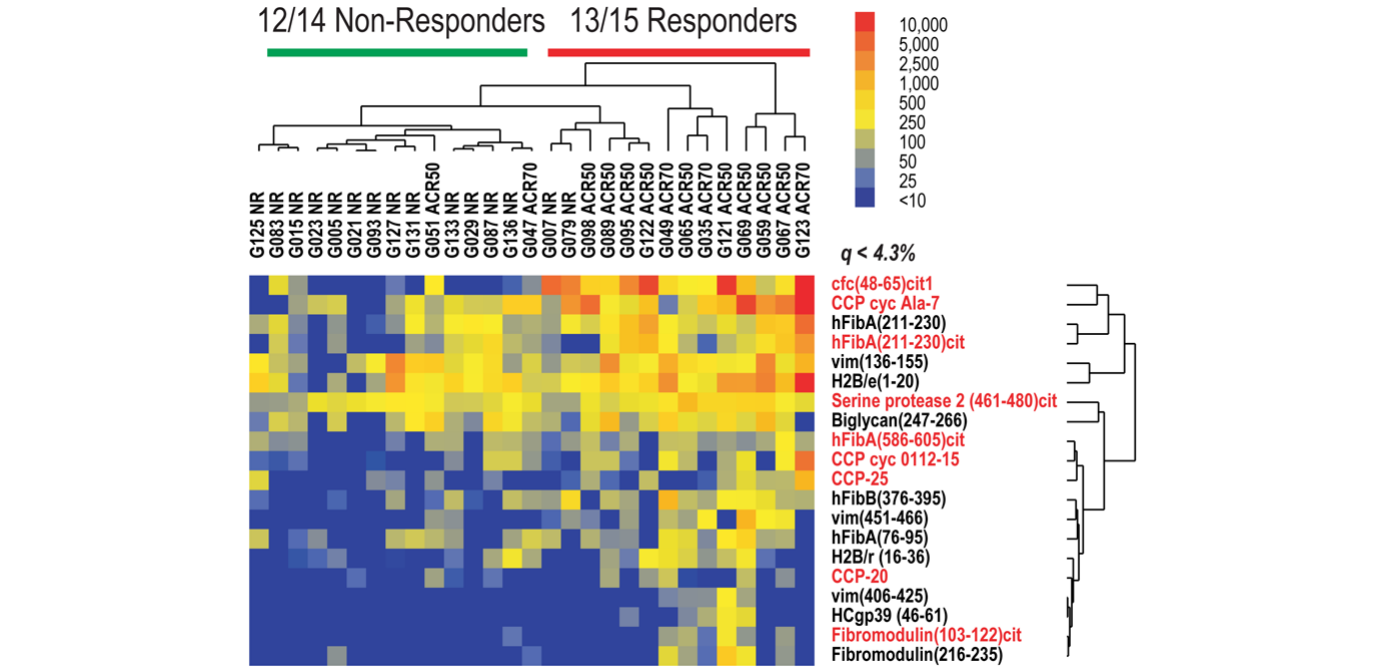
Elevated pretreatment autoantibody profiles in etanercept responders compared with nonresponders in the ABCoN cohort. Significance analysis of microarrays (SAM) and hierarchical clustering were applied to identify and display autoantibody profiles that differentiate etanercept responders from nonresponders; results from one of several representative experiments are presented. SAM was utilized to identify antigens with statistical differences in antibody reactivity between etanercept responders (≥ ACR50) and nonresponders (< ACR20), and the statistically significant hits are listed to the right of the heatmap (false discovery rate *q *< 4.3%). The SAM-identified variables and individual patients were then hierarchically clustered, and results presented in tree dendrograms that represent the relationships in reactivities between patients as well as between antigens. Red font, citrullinated antigens; black font, native antigens. Patients are listed across the top of the heatmap image, and the ACR response rate for each patient is indicated. Red bar, responder cluster; green bar, nonresponder cluster. Numbers of misclassified samples are shown for each cluster. Array fluorescence units are color coded and indicated in the bar in the right upper corner of the image. ACR, American College of Rheumatology response; CCP, cyclic citrullinated peptide.

We screened 29 pretreatment serum samples from the ABCoN cohort using RA antigen microarrays. SAM identified differential pretreatment levels of autoantibodies that were present at elevated levels in etanercept responders (response ≥ ACR50) as compared with nonresponders (response, < ACR20). A representative result of the top-scoring SAM-identified autoantigens (false discovery rate (*q *values) ≤ 4.3%) is presented in Figure 3, and hierarchical cluster analysis was performed to organize and visualize relationships between samples and antigens (Cluster^® ^software). A compiled list of the top-scoring antigens identified in multiple screening experiments is presented in Table 2. The SAM-identified top-scoring antigens were overlapping but were not completely identical between array screening experiments. While all of the antigens presented in Table 2 were identified as top scorers in multiple array experiments, a few were ranked below the threshold for the top-scoring antigens identified in the experiment used to generate the representative cluster shown in Figure 3.

#### Cytokine profiling using a bead-array system

We performed multiplex cytokine profiling using the Luminex bead array system and methods previously optimized to minimize the impact of rheumatoid factor [12]. Our analysis of 12 cytokines in the initial 29 ABCoN samples characterized did not reveal significant differences between responders and nonresponders based on both linear regression as well as SAM and PAM analysis, probably due to a substantial number of patients with very low or undetectable levels of many of the cytokines (data not shown). When 64 further samples from etanercept-treated patients from two additional cohorts became available, and cytokine profiling results from this larger set of samples were analyzed by regression analysis, however, significant differences in baseline cytokine levels were identified in responders (response ≥ ACR50) as compared with nonresponders (response ≤ ACR20). These results are presented in detail below.

#### Combinations of autoantibody and cytokines improve differentiation of etanercept responders from nonresponders

To determine whether combinations of cytokines and autoantibodies might provide superior differentiation of pretreatment samples derived from etanercept responders and nonresponders, we next performed SAM analysis on integrated antigen array and cytokine datasets obtained for the 29 ABCoN samples. This analysis demonstrated that a panel of antigens and cytokines more effectively differentiated baseline samples derived from responders and nonresponders (*q *< 3; data not shown). Based on these preliminary observations, combined autoantibody and cytokine analyses were used in the subsequent experiments outlined below with the objective to develop a multi-parameter biomarker for predicting response to etanercept therapy.

### Autoantibody and cytokine profiling of pretreatment samples derived from three cohorts

In the next series of experiments, we utilized pretreatment samples from three independent cohorts of etanercept new-start RA patients. These cohorts included the US-based ABCoN cohort, a Swedish cohort, and a Japanese cohort.

#### Analysis of cytokines

In a first step, concentrations of 12 cytokines were measured and analyzed by logistic regression in all 93 pretreatment samples derived from the three independent cohorts. When cytokine results from baseline samples derived from responders and nonresponders were compared, TNF and IL-15 were elevated in responders as compared with nonresponders (*P *< 0.05), while MCP-1 and IL-6 exhibited a trend towards being elevated in responders as compared with nonresponders (*P *< 0.1).

**Figure 4:**
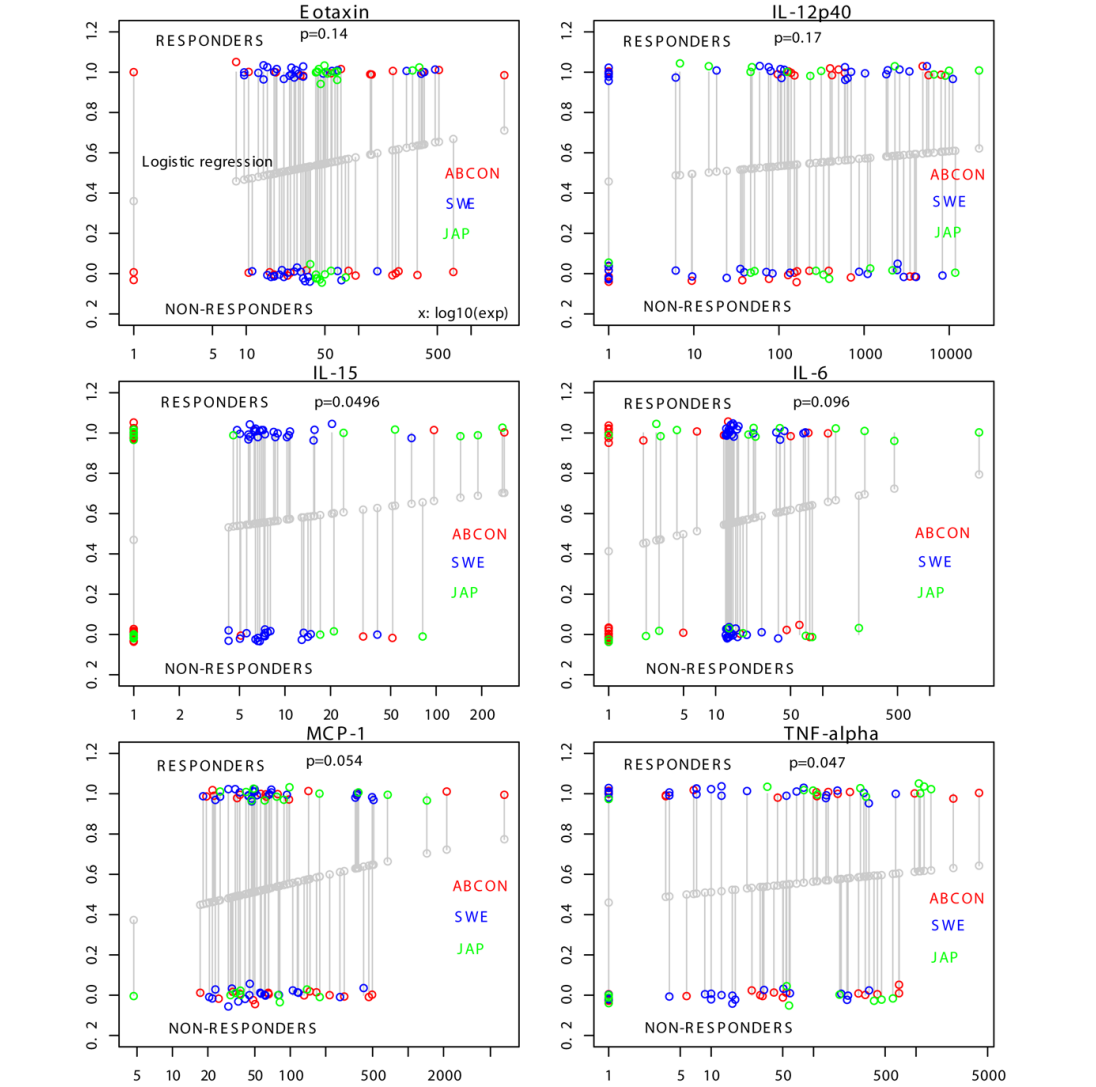
Elevated pretreatment blood cytokines are associated with response to etanercept therapy. Logistic regression analysis was applied to cytokine measurements in 93 samples derived from three cohorts of etanercept new-start rheumatoid arthritis patients. Green circles, samples from the Japanese cohort; red circles, samples from the ABCoN cohort; blue circles, samples from the Swedish cohort; grey circles, the best-fit logistic regression curve; *x *values, actual cytokine concentrations; *y *values, an artificial noise value was added to achieve better visual separation of the actual cytokine values. *P *values are shown for each cytokine. The grey bar links the actual responder or nonresponder label of a sample with the logistic regression result of the same sample (probability of being a responder). For better readability, only six cytokines are shown. MCP-1, monocyte chemoattractant protein-1.

To visualize results from logistic regression analysis of cytokines from all three cohorts, Figure 4 presents trendlines for six of the 12 cytokines in all three cohorts. The grey dots in Figure 4 demonstrate the best-fit logistic regression curve. The *x *values indicate cytokine concentrations, while in the *y *dimension an artificial noise value was added to achieve better visual separation of the actual cytokine values. Overall, the trends for all analyzed cytokines showed higher baseline serum concentrations in the responders as compared with the nonresponders (grey trendlines in all panels of Figure 4; see also Additional data file 3).

The classification error rates, however, were determined to be 39.8% to 48.4%; thus, taken alone, pretreatment blood cytokine levels appear to be of no practical utility in classifying the likelihood for response to etanercept therapy. We concluded that only in combination with other biomarkers do pretreatment blood cytokine concentrations contribute to a predictive biomarker signature for response to etanercept.

#### Analysis of autoantibodies only

Based on the initial RA antigen array experiments in the ABCoN cohort described above, to further test candidate antibody biomarkers with the greatest predictive utility we developed peptide ELISAs for the most promising peptide antigens. All 93 pretreatment samples from etanercept new-start patients were analyzed with these ELISAs, and the relative autoantibody measurements optical density (OD) values were used for further analyses. Since relative expression levels were the primary measure of interest for these assays, no standard curves for calculation of antibody concentrations were developed. All measurements from the single-antigen ELISAs were combined with the measurements from the bead-array cytokine assay for integrated analysis of cytokine and autoantibody profiles in baseline samples derived from all three cohorts of etanercept new-start RA patients.

#### Combined autoantibody and cytokine profiles are most predictive for response to etanercept in three independent cohorts

**Figure 5:**
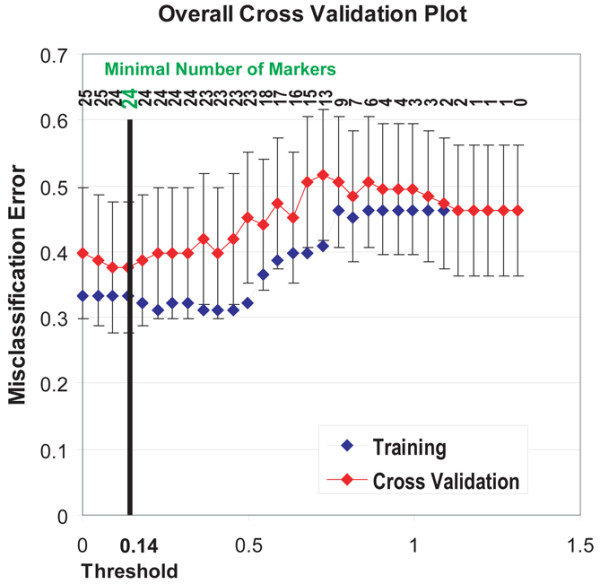
Identification of a 24-antibody and cytokine biomarker that differentiates pretreatment etanercept responders from nonresponders. Prediction analysis of microarrays (PAM) was applied to establish a rank list of the variables, by training PAM on all 93 samples. An overlay of error plots derived from PAM analysis of the 93 samples is displayed. First, PAM was trained on the multi-parameter biomarker; blue, training error graph. Second, internal cross-validation of the dataset was performed; red, overall error of the cross-validation. For better readability, error bars are shown for the cross-validation graph only. The number of markers is shown in ascending order from right to left across the top of the panel, and the selected PAM-derived threshold is indicated.

**Figure 6:**
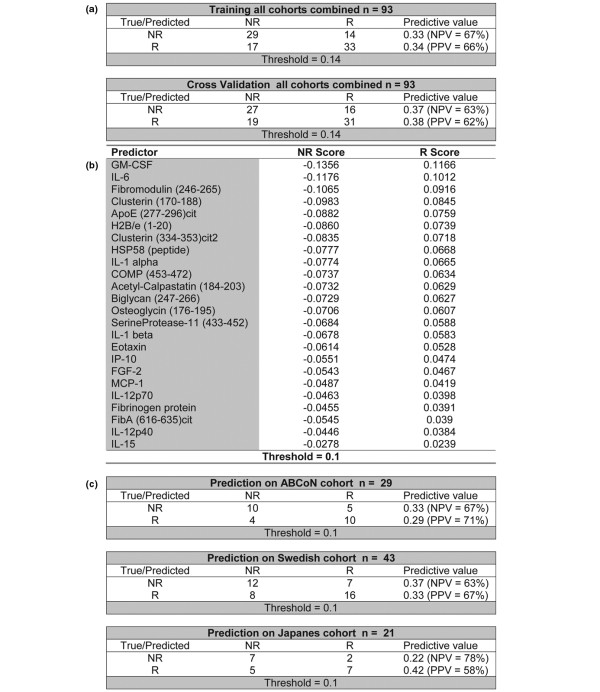
Prediction of responders and nonresponders. Calculations of classification errors for **(a) **the PAM training/cross-validation step on all cohorts combined, and **(c) **the PAM prediction steps for the three cohorts individually (top panel, ABCoN cohort; middle panel, Swedish cohort; bottom panel, Japanese cohort). R, responder; NR, nonresponder; NPV, negative predictive value; PPV, positive predictive value. **(b) **Complete biomarker of 24 discriminators listed according to rank order, with associated scores for nonresponders and responders in the right and far-right columns, respectively. ApoE, apolipoprotein E; COMP, cartilage oligomeric matrix protein; FGF-2, fibroblast growth factor-2; GM-CSF, granulocyte- macrophage colony-stimulating factor; IP-10, IFNγ-inducible protein 10; MCP-1, monocyte chemoattractant protein-1.

PAM with error plots of training and cross-validation are shown in Figure 5. To illustrate their parallelism, the graphs for training and cross-validation were overlaid and presented in one image. A threshold was determined that allowed optimal segregation of the pretreatment samples derived from responders and nonresponders; the prediction threshold that enabled best differentiation with the minimal number of predictors was identified to be 0.14 (*T*_(0.14)_) (Figure 5, vertical bar). Twenty-four parameters were included in the signature at *T*_(0.14)_, and the PAM rank list of these parameters including their corresponding scores for responders and nonresponders are shown in Figure 6b. The list comprised 13 antigens and 11 cytokines.

To further test the biomarker signature identified at *T*_(0.14)_, and to determine its utility for prediction of response in each cohort independently, the 24-parameter classifier was then applied to the three cohorts individually. Prediction probabilities were calculated for the responder and nonresponder classes. Classification errors as well as positive predictive values and negative predictive values are shown in the corresponding tables for each cohort (Figure 6c). In summary, the positive predictive value ranged from 58% (Japanese cohort) to 72% (ABCoN), and the negative predictive value ranged from 63% (Swedish cohort) to 78% (Japanese cohort).

## Discussion

We describe the proteomic screening and discovery of a 24-biomarker signature in pretreatment samples derived from RA patients for class prediction of response to therapy with the anti-TNF therapy etanercept. We developed and tested the signature using three independent population-based cohorts from the USA, Sweden and Japan. Our results indicate that the 24-variable biomarker has utility to predict good to excellent response to etanercept therapy (equivalent to response ≥ ACR50 for the ABCoN and Swedish cohorts, and to response ≥ ACR70 for the Japanese cohort), and to predict lack of response to etanercept therapy (response < ACR20). This biomarker signature enabled superior pretreatment classification of response in three ethnically diverse cohorts, in comparison with a theoretical benchmark based on clinical observation and previous experience in population-based cohorts (one-third of patients no response, one-third of patients partial response, and one-third of patients good response).

Identification of clinical predictors and development of molecular biomarkers have been hampered by many factors, including the molecular complexity and clinical heterogeneity of RA, the inherent difficulty in classifying response to therapy that appears random and does not follow a Gaussian distribution [20], and the lack of enabling technologies to broadly screen for potential biomarkers. Several single-cohort studies reported associations of acute phase parameters [21], genetic factors [22], Fcγ receptor type IIIA polymorphisms [23], – 308 TNFα gene polymorphisms [24], and rheumatoid factor or anti-citrulline autoantibody titers [25] with response to anti-TNF therapies. Nevertheless, studies investigating multiple cohorts using proteomic-scale biomarker signatures have not yet been reported. Although providing great potential, genomic and proteomic profiles identified in single cohorts of patients have frequently failed to replicate when subsequently applied to independent cohorts [26].

To address this unmet clinical need and some of the above-mentioned limitations, we applied proteomics technologies to characterize pretreatment samples from three independent cohorts, whereby all patients were treated with a single anti-TNF therapy (etanercept).

Using data from all three cohorts, we identified a panel of proteins, characterized by elevations of both serum antibody and cytokine concentrations, which were associated with patients who exhibited clinically good response (≥ ACR50) and excellent response (≥ ACR70) to etanercept therapy (positive predictive value = 58 to 71%). In contrast, patients who exhibited minimal or no significant response to etanercept therapy after 3 months or more were found to predominantly lack this biomarker signature (response < ACR20; negative predictive value = 63 to 78%).

The RADIUS program – the Rheumatoid Arthritis DMARD Intervention and Utilization Study [8], which follows two multicenter observational registries of thousands of RA patients from rheumatology practices – demonstrated that ACR response rates were notably lower as compared with the sentinel clinical trials. This observation might suggest that the benchmark for novel biomarkers to predict response in general rheumatology practice could be different from the benchmark for clinical trials. It is possible that, with further refinement and use of Good Laboratory Practice assays, autoantibody and cytokine profiling could provide significantly higher predictive values for predicting response to anti-TNF therapies. A biomarker that increases the precision of pretreatment prediction of the ACR50 response (that is, from 50 to 70%) provides the potential to have a significant impact on clinical decision-making and potentially trial design [27].

Our study investigated samples from three population-based cohorts. Within the US-based ABCoN cohort, for which the multi-parameter biomarker demonstrated the best response prediction, analysis of the responders exhibiting an ACR50 response or greater provided a positive predictive value of 71% (Figure 6c). In the Japanese cohort, the level of positive prediction observed was lower (positive predictive value = 58%), while the biomarker identified Japanese nonresponders with a negative predictive value of almost 80%, which was the highest of any predictive value seen in this study.

Some of the markers in the 24-variable biomarker signature were previously proposed to have some value for prediction, such as MCP-1 [28] and titers of autoantibodies targeting certain citrullinated antigens or mixtures thereof, as measured in the cyclic citrullinated peptide ELISAs [29,30]. Our data corroborate these earlier notions since several citrullinated peptides emerged as useful variables, as well as MCP-1 demonstrating a near-significant trend of being elevated in responders. Newly described markers include several native and nonfilaggrin-derived citrullinated antigen epitopes as presented in Table 2. Our data also demonstrate that cytokines alone are probably insufficient to accurately predict treatment responses in a general population, and combinations of autoantibodies and cytokines provide increased predictive utility across ethnically diverse and clinically heterogeneous RA populations. Additional markers (imaging, genetic, clinical) may eventually have to be incorporated into a definitive panel of biomarkers [31], for the future development of a prediction score that could be applied for reliable patient stratification.

Although the present study utilizes samples from three cohorts of patients, important limitations remain: the number of samples per cohorts is relatively small; a number of samples demonstrated undetectable serum levels of cytokines; the quantification of response to therapy in RA is intrinsically inaccurate since response classification is based on short term follow-up; and our data may underestimate the predictive value of these biomarkers for predicting response to anti-TNF therapy since concomitant disease-modifying anti-rheumatic drug medication in the three cohorts was not uniform. Moreover, a full *validation *in independent cohorts of the identified markers is not presented in this paper. Because of the small sample numbers in the individual cohorts and the fact that the experiments were performed over a 3-year period, the present article represents a discovery and testing study. The training was performed on the full dataset, and the resulting panel of markers was tested in the three individual cohorts. These limitations indicate the need for larger validation studies and prospective serum proteome studies in cohorts where longer-term response outcomes are available.

## Conclusions

We identified a 24-parameter autoantibody and cytokine biomarker that enables pretreatment classification and prediction of etanercept responders in three cohorts of patients with RA. Although further validation in prospective and larger cohorts is needed, it is proposed that the multi-parameter protein biomarker presented in this study will facilitate the development of molecular biomarkers with even higher predictive utilities for guiding use of anti-TNF therapy and other disease-modifying therapies in RA patients.

## Abbreviations

ACR: American College of Rheumatology; COMP: cartilage oligomeric matrix protein; ELISA: enzyme-linked immunosorbent assay; IL: interleukin; MCP-1: monocyte chemoattractant protein-1; PAM: prediction analysis of microarrays; PBS: phosphate-buffered saline; RA: rheumatoid arthritis; SAM: significance analysis of microarrays; TNF: tumor necrosis factor.

## Competing interests

The authors declare that US and international patent applications were filed for biomarkers to guide selection of therapy in RA, and option agreements for this intellectual property have been signed with third-party companies.

## Authors' contributions

WH and WHR conceived of the study and designed the arthritis antigen arrays. PAM performed immunoprecipitation experiments and mass spectrometry studies. WH and BHT carried out the microarray experiments, and multiplex cytokine and ELISA experiments. RJT, WL and BHT performed the statistical analysis. FB, RFvV, JL, LK, YT, KS, PKG and MCG participated in design and coordination. WH drafted the manuscript. All authors read and approved the final manuscript.

## Supplementary Material

Additional data file 1 Excel file containing a table that summarizes the clinical characteristics of the Japanese cohort, including co-medications. Click here for file

Additional data file 2 Adobe Illustrator file containing a figure that shows the analysis of microarray reactivities by conventional ELISA. Array-determined digital fluorescence units (arrayFU) are plotted on the *x *axis and optical density values are plotted on the *y *axis. Coefficients of linear regression analysis (*R*^2^) are shown for each peptide. Click here for file

Additional data file 3 Adobe Illustrator file containing a figure that demonstrates logistic regression analysis of the six other cytokines not shown in Figure 4. See Figure 4 for details. Click here for file
