# Supplementary figures and images for: Blood autoantibody and cytokine profiles predict response to anti-tumor necrosis factor therapy in rheumatoid arthritis
Source: Arthritis Res Ther. 2009 May 21;11(3):R76. doi: 10.1186/ar2706 (PMC2714123; doi:10.1186/ar2706)

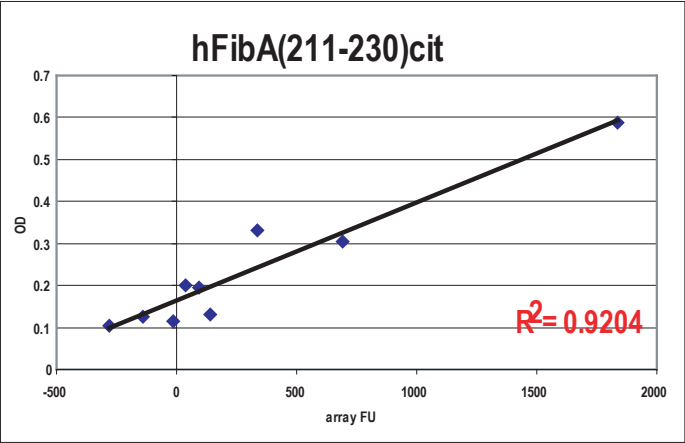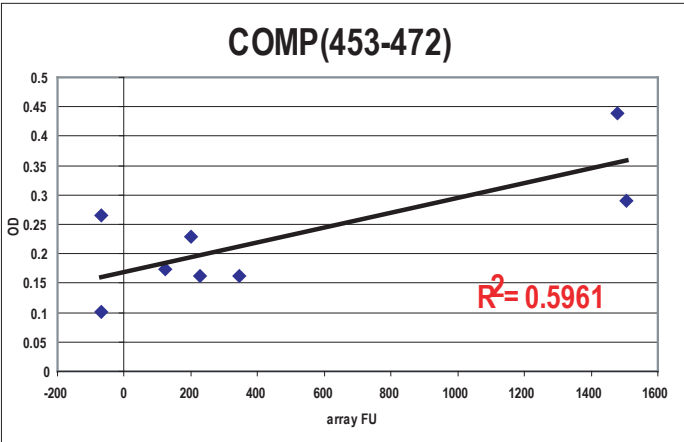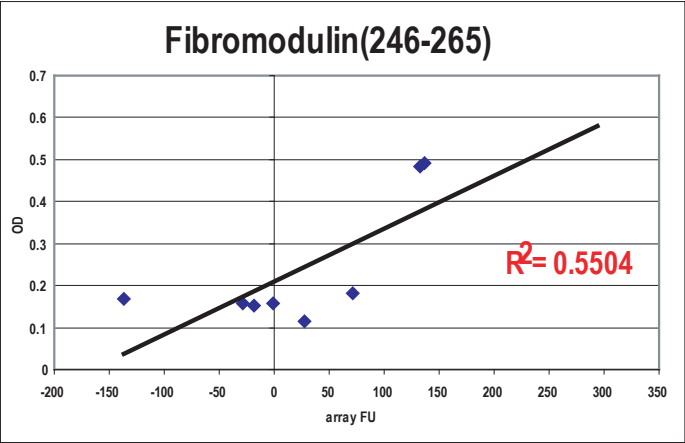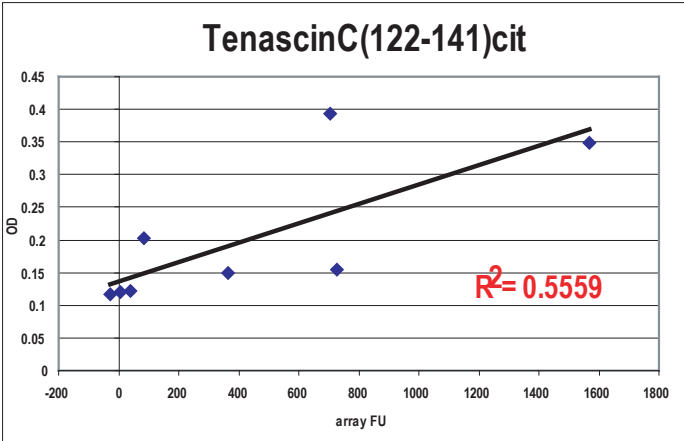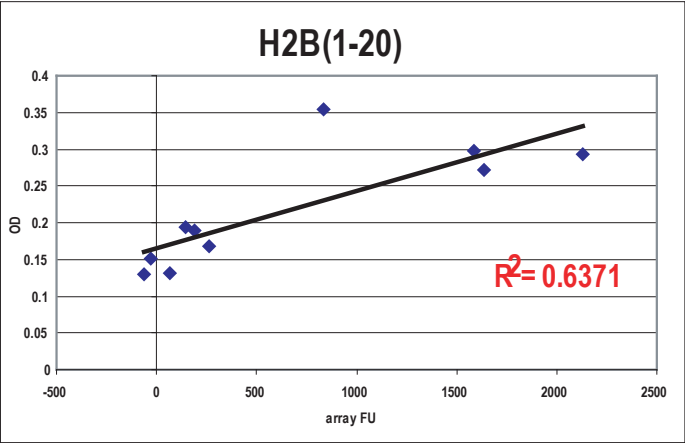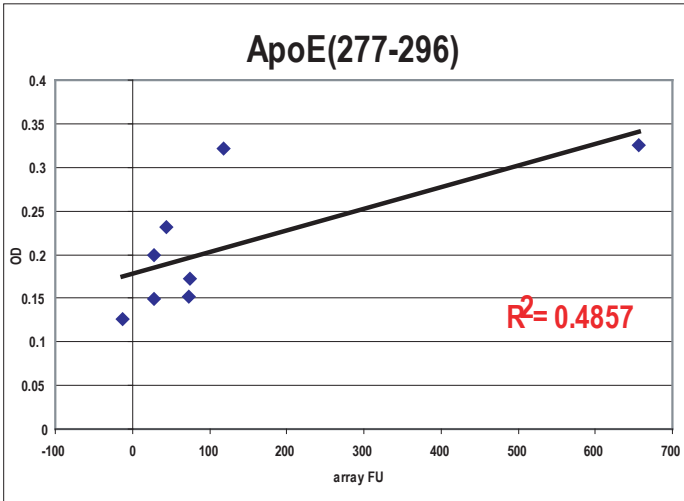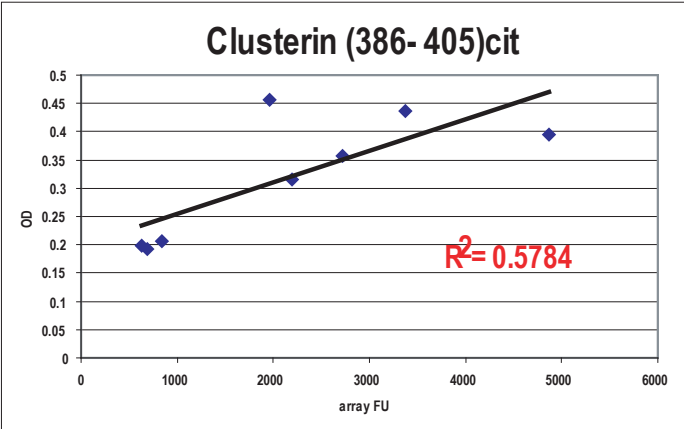

Supplement: Additional data file 2 — Adobe Illustrator file containing a figure that shows the analysis of microarray reactivities by conventional ELISA. Array-determined digital fluorescence units (arrayFU) are plotted on the x axis and optical density values are plotted on the y axis. Coefficients of linear regression analysis (R2) are shown for each peptide. [file ar2706-S2.pdf]

## Additional data file 3

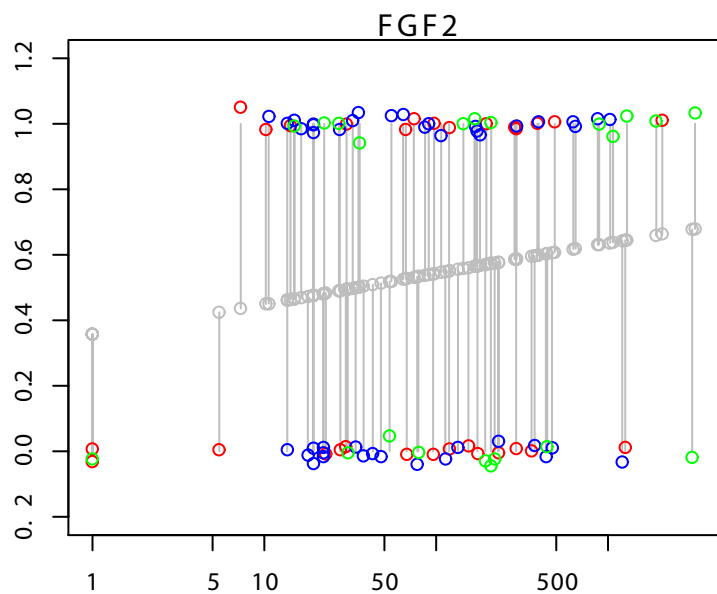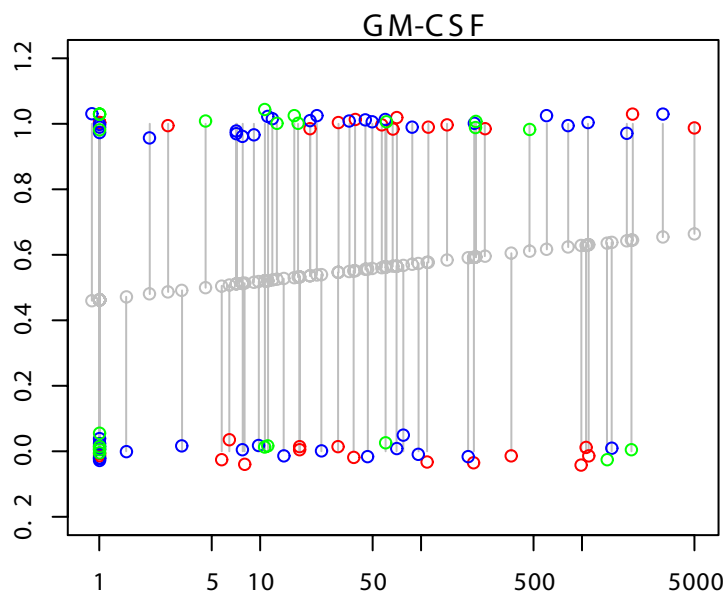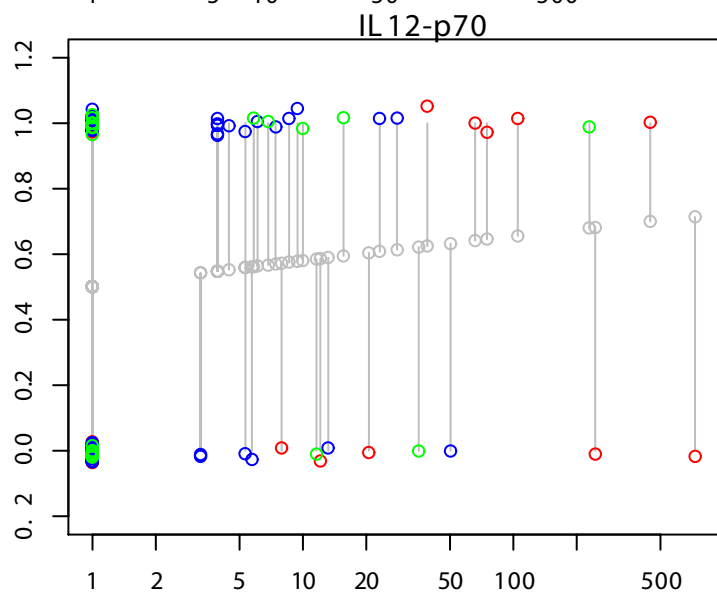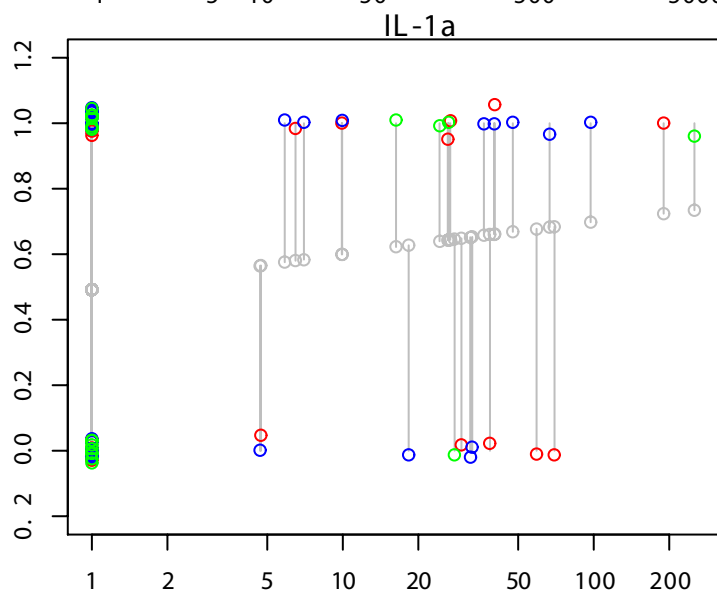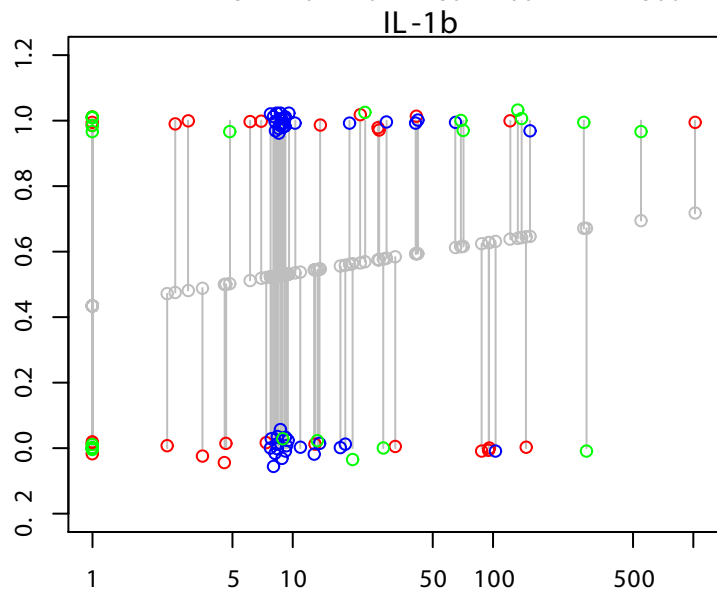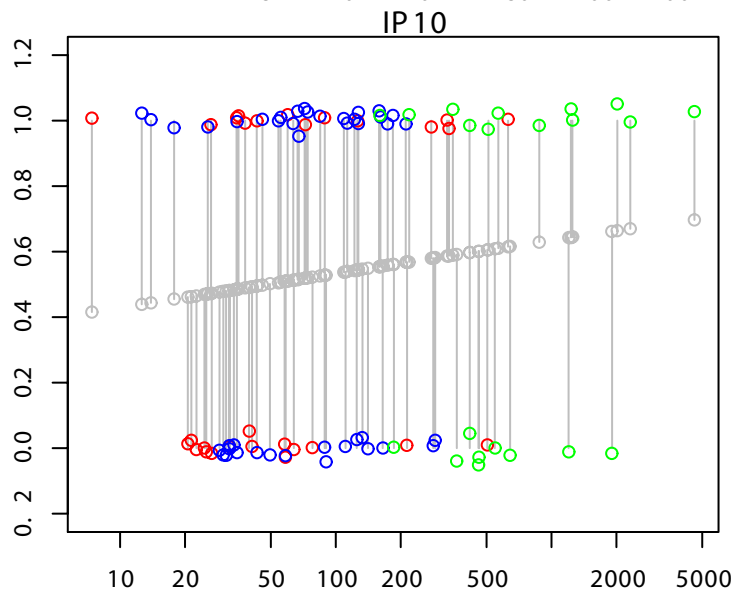

Supplement: Additional data file 3 — Adobe Illustrator file containing a figure that demonstrates logistic regression analysis of the six other cytokines not shown in Figure 4. See Figure 4 for details. [file ar2706-S3.pdf]
